# Peroxiredoxin 2 is highly expressed in human oral squamous cell carcinoma cells and is upregulated by human papillomavirus oncoproteins and arecoline, promoting proliferation

**DOI:** 10.1371/journal.pone.0242465

**Published:** 2020-12-17

**Authors:** Jureeporn Chuerduangphui, Tipaya Ekalaksananan, Chukkris Heawchaiyaphum, Patravoot Vatanasapt, Chamsai Pientong

**Affiliations:** 1 Department of Microbiology, Faculty of Science, Kasetsart University, Bangkok, Thailand; 2 HPV & EBV and Carcinogenesis Research Group, Khon Kaen University, Khon Kaen, Thailand; 3 Department of Microbiology, Faculty of Medicine, Khon Kaen University, Khon Kaen, Thailand; 4 Department of Otorhinolaryngology, Faculty of Medicine, Khon Kaen University, Khon Kaen, Thailand; Duke University School of Medicine, UNITED STATES

## Abstract

Peroxiredoxin 2 (PRDX2) is upregulated in various cancers including oral squamous cell carcinoma (OSCC). It is a known tumor promoter in some cancers, but its role in OSCC is unclear. This study aimed to investigate the effect of arecoline, an alkaloid of the betel nut, and human papillomavirus type 16 (HPV16) E6/E7 oncoproteins on induction of PRDX2 expression, and also the effects of PRDX2 overexpression in oral cell lines. Levels of PRDX2 protein were determined using western blot analysis of samples of exfoliated normal oral cells (n = 75) and oral lesion cells from OSCC cases (n = 75). Some OSCC cases were positive for HPV infection and some patients had a history of betel quid chewing. To explore the level of PRDX2 by western blot, the proteins were extracted from oral cell lines that were treated with arecoline or retroviruses containing HPV16 E6 gene and HPV16 E6/E7 expressing vector. For analysis of PRDX2 functions, cell proliferation, cell-cycle progression, apoptosis and migration was compared between oral cells overexpressing PRDX2 and cells with PRDX2-knockdown. PRDX2 expression levels tended to be higher in OSCC samples that were positive for HPV infection and had history of betel quid chewing. Arecoline treatment *in vitro* at low concentrations and overexpression of HPV16 E6 or E6/E7 in oral cells induced PRDX2 overexpression. Interestingly, in oral cells, PRDX2 promoted cell proliferation, cell-cycle progression (G2/M phase), cell migration and inhibited apoptosis. Upregulation of PRDX2 in oral cells was induced by arecoline and HPV16 oncoproteins and promoted growth of OSCC cells.

## Introduction

Peroxiredoxins (PRDXs) are a group of thiol-dependent peroxidases that control various biological processes as well as tumorigenesis. Therefore, the level of PRDXs in many human cancers have studied both *in vitro* or *in vivo* models [1]. Moreover, PRDXs promote the cancer cell stemness, chemoresistance and cancer aggregation [2].

PRDX2 plays a role in reduction of reactive oxygen species (ROS), such as hydrogen peroxide (H_2_O_2_). Interestingly it regulates multiple cellular functions, including cell proliferation, differentiation and intracellular signaling [3, 4]. PRDX2 is upregulated (and therefore could be a biomarker) in various cancers including colorectal, renal, breast and lung cancers [5–7]. Inversely, PRDX2 functions as a tumor suppresser in acute myeloid leukemia [8]. However, the function of PRDX2 in oral carcinogenesis remains unclear and the mechanism leading to upregulation of PRDX2 in cancer, particularly in oral squamous cell carcinoma (OSCC), has not been explored.

Expression of PRDX2 can be induced by many mechanisms, particularly oxidative stress, which causes dissociation of the transcription factor nuclear factor erythroid-2-related factor 2 (Nrf2) from Keap1/INrf2 [9–11]. Consequently, Nrf2 enters the nucleus and binds to a specific cis-acting antioxidant responsive element (ARE) in the *PRDX2* gene. Expression of PRDX2 is then elevated to attenuate oxidative stress [12]. Additionally, oxidative stress also plays roles in carcinogenesis in various malignancies including OSCC [13]. In addition, hypoxia-inducible factors HIF-1α and HIF-2α are rapidly expressed in hypoxia and then induce PRDX2 expression [14]. Even though there are many reports demonstrating the regulation of PRDX2, the OSCC-associated factors including areca nut/betel quid chewing, smoking, alcohol consumption and HPV infection on this have not been investigated [12, 14–16]. This study, therefore, has evaluated the effect of betel quid chewing and HPV on PRDX2 expression in oral specimens and cell lines because both factors are the major risk of OSCC in Northeast Thailand region.

Human papillomavirus (HPV) is an independent risk factor for OSCC [17]. High-risk HPV oncoproteins, E6 and E7, play important roles in tumorigenesis via their aberrant downstream cascades [18]. The effect of HPV oncoproteins on PRDX2 upregulation in OSCC is unknown. However, there have been reports that HPV E2 and E6 can regulate oxidative stress [19, 20]. Moreover, HPV E6 also induces HIF-1α [21]. Areca nut/betel quid chewing is well known to be a major risk factor for OSCC, particularly in Asian populations [22]. Arecoline is a major constituent of areca nut and can induce oxidative stress and HIF-1α expression [23, 24]. Hypothetically, both factors may promote oral carcinogenesis by regulating PRDX2 via various mechanisms.

## Materials and methods

### Clinical samples

Exfoliated normal oral cells from the buccal mucosa of cancer-free controls (75 individuals) and oral lesion cells from OSCC cases (75 individuals) were collected by brushing. They were initially evaluated and/or diagnosed by an otorhinolaryngologist at the Department of Otorhinolaryngology, Srinagarind Hospital, Faculty of Medicine, Khon Kaen University. This study was conducted under the approval of Khon Kaen University Ethics Committee for Human Research (HE561407 and No. HE601490). Informed consent was obtained in writing from all participants prior to collect samples.

### Cell lines and culture

Human OSCC cell lines, ORL-48T and ORL-136T, were kindly provided by Prof. Sok Ching Cheong (Cancer Research Initiatives Foundation, Sime Darby Medical Centre Jaya, Malaysia). HeLa, human embryonic kidney (HEK) 293T and human tongue keratinocyte (HTK1-K4DT and HTK1-K4DT-16E6SD) cell lines were kindly provided by Prof. Tohru Kiyono (National Cancer Center Research Institute, Japan). Cells were maintained in DMEM/F12, DMEM and F-media media (Gibco, Grand Island, NY, USA) containing 10% fetal bovine serum (HiMedia, India) at 37°C in a humidified incubator.

### Treatment of oral cells with arecoline

Cells of HTK1-K4DT or ORL-48T were seeded into 24-well plates (150,000 cells per well) and incubated for 24 hours. Various concentrations (0, 0.01, 0.025, 0.25, 2.5 and 25 μg/mL) of arecoline (Sigma-Aldrich, St. Louis, MO, USA) were used to treat each cell line and maintained for 24 and 48 hours and then PRDX2 mRNA and protein levels were evaluated. Concentrations of arecoline used are those described in our previous study [25].

### Construction of pIRES2-EGFP vector containing HPV16 E6/E7 fragment

The HPV16 E6/E7 fragment from pLXSN-16E6/E7 vector was digested with *Eco*RI and *Bam*HI enzymes and subcloned into a pIRES2-EGFP vector. A vector containing the correct fragment was identified using PCR and confirmed by sequencing analysis as described in a previous study [15]. The pIRES2-EGFP and pIRES2-16E6/E7 plasmids were extracted and purified using EZNA™ plasmid mini prep kit (Omega Bio-tek, Doraville USA). Quantity and quality of the plasmid DNA were determined using a NanoDrop ND-2000c (Thermo Scientific).

### Transfection of pIRES2-EGFP and pIRES2-16E6/E7 vectors into oral cell lines

HTK1-K4DT and ORL-48T cells were seeded into 24-well plate at 150,000 cells per well. The cells were transfected with pIRES2-EGFP or pIRES2-16E6/E7 vector (500 ng/well) using Lipofectamine 2000 (Invitrogen) for 6 hours. After 24 and 48 hours post-infection, total RNA and protein were extracted using TRIzol® Reagent (Invitrogen Corp., Carlsbad, CA, USA). The extracted RNA was used to synthesize cDNA by RevertAid™ H Minus First Strand cDNA Synthesis Kit (Fermentas, Ontario, Canada).

### Production of retrovirus carrying the pCLXSN-PRDX2 vector

Plasmid containing the PRDX2 gene was constructed using human PRDX2 cDNA from ORL-48T cells that was amplified according to S1 Table, and then cloned into pDORN221 vector and switched into pCLXSN vector (kindly provided by Prof. Tohru Kiyono) by recombinational cloning using Gateway BP and LR clonases (Invitrogen), respectively. HEK 293T cells were seeded into 10-mm culture dishes at 5 x 10^6^ cells/dish and incubated at 37°C for 24 hours. pCLXSN-PRDX2 and retroviral packaging vectors (pCL-Gag/Pol and pHCMV-VSV-G) were co-transfected into HEK 293T cells overnight. The medium was collected 48 and 96 hours post-transfection. The colony-forming unit assay with HeLa cells was performed to determine the viral titer [26].

### Establishment of PRDX2-overexpressing ORL-48T cells

ORL-48T cells were seeded into 24-well plates at 100,000 cells per well and transduced by retrovirus-carrying pCLXSN-PRDX2 vector at MOI 1. After 48 hours, medium containing 200 μg/mL of G418 was used to select resistant clones of transduced ORL-48T cells for 14 days. The retrovirus-carrying pCLXSN vector acts as a control.

### Determination of PRDX2 and GAPDH expression

cDNA was synthesized using RevertAid First Strand cDNA Synthesis Kit (Thermo Scientific, USA) and was added to Sso-Advanced SYBR Green Supermix (Bio-Rad, Hercules, CA, USA) including 0.3 mM of each primer (S1 Table) and 20 ng of DNA template. Real-Time PCR was carried out to detect expression of PRDX2 and GAPDH using the CFX96 Real-Time PCR Detection System (Bio-Rad, Hercules, CA, USA). The relative quantification (RQ) of gene expression was determined using the 2^-ΔΔCT^ approach.

### Protein extraction from oral cell samples and oral cell lines

The oral cell samples and trypsinized cell culture were washed twice with 1xPBS. The cell pellet was lysed by TRIzol® Reagent according to manufacturer's protocol. Briefly, 500 μL of Trizol® Reagent was added to the cell pellet followed by 100 μL of chloroform and vigorous mixing. After incubation on ice for 15 minutes, the layers were separated by centrifugation at 12,000 rpm for 15 minutes. The upper layer was removed to extract RNA. The lower phase was collected and ethanol added to precipitate DNA and then centrifuged at 2,000 rpm. The supernatant was collected to precipitate protein by additional isopropanol. The protein pellet was denatured using guanidine hydrochloride and washed using ethanol. Finally, dried protein was dissolved in rehydration buffer (8 M urea and 2% CHAPS).

### Western blot

Protein concentrations (from oral cell samples, arecoline-treated/untreated cell lines, transfected/untransfected cells, and transduced cells) were measured relative to a bovine serum albumin (BSA) calibration curve using the Bradford reagent (Bio-Rad, Richmond, CA, USA). PRDX2 and beta-actin proteins were determined by western blot, using as primary antibodies mouse anti-PRDX2 (1:2,000 dilution; clone 1E8, ab188290, Abcam, USA) and mouse anti-beta-actin (1:1,000 dilution; clone C4, sc-47778, Santa Cruz Biotechnology, Inc, Santa Cruz, Cal, USA), respectively. IgGκ binding protein-HRP (m-IgGκ BP-HRP) (sc-516102, Santa Cruz Biotechnology) was used as the secondary antibody. The signal was produced using ECL Prime Western Blotting Reagent (GE). The intensity of protein bands was measured using Image J 1.49v software (National Institutes of Health, Bethesda, MD, USA).

### Cell proliferation in PRDX2-overexpressing and siRNA knockdown

PRDX2-overexpressing ORL-48T cells were seeded into 96-well plates at 10,000 cells per well and maintained in DMEM/F12 medium supplemented with 10% FBS and antibiotics for 24, 48 and 72 hours. Ten microliters of MTT (5 mg/mL) was then added to each well. After 4 hours, the medium was removed and the water-insoluble purple formazan particles were dissolved in 100 μL DMSO solution. The absorbance was read at 540 nm with a Microplate Reader (TECAN, Salzburg, Austria).

For PRDX2-knockdown, each HTK1-K4DT, ORL-48T and ORL-136T cell line was seeded into 96-well plates at 10,000 cells per well. Thereafter, cells were transfected using Lipofectamine 2000 with either 100 nM siRNA-Control (AM4611, Ambion, Austin, TX, USA) or 100 nM siRNA-PRDX2 (HSS186275, Invitrogen) according to manufacturer’s instructions. At 48 hours post-transfection, cell population was measured using the MTT assay as mentioned above.

### Cell apoptosis in PRDX2-overexpressing cells and siRNA knockdown

PRDX2-overexpressing ORL-48T cells were seeded into 6-well plates at a density of 200,000 cells per well. After incubation for 24 hours, the medium was changed to DMEM/F12 containing 2.5% FBS and antibiotics and maintained for 72 hours. These cells were then collected by trypsinization and washed twice with PBS. After washing, the cells were suspended in Annexin-binding buffer and then stained with an Invitrogen™ Dead Cell Apoptosis Kit with Annexin V FITC and PI (Thermo Fisher Seintific Inc., Germany). After 15-minute incubation, the cells were analyzed using a FACSCanto flow cytometer (Becton–Dickinson).

For PRDX2-knockdown, ORL-48T cells were seeded into 24-well plates at 200,000 cells per well and maintained in DMEM/F12 medium supplemented with 2.5% FBS and antibiotics. Thereafter, cells were transfected using Lipofectamine 2000 with either 100 nM siRNA-Control or 100 nM siRNA-PRDX2 according to manufacturer’s instructions. Sixteen hours after siRNA transfection, the medium was changed to DMEM/F12 containing 2.5% FBS and maintained for 72 hours as above.

### Cell-cycle progression in PRDX2-overexpressing cells and siRNA knockdown

PRDX2-overexpressing ORL-48T cells were seeded into 24-well plates at a density of 150,000 cell per well. After incubation for 24 hours, the medium was changed to DMEM/F12 containing 2.5% FBS and antibiotics and maintained for 72 hours. These cells were then collected by trypsinization, washed twice with cold PBS and fixed in 70% ethanol for at least 16 hours. After washing, propidium iodide (PI) solution (BD Pharmingen, Heidelberg, Germany) was used to stain the cells. After a 15-minute incubation, the cells were analyzed using a FACSCanto flow cytometer (Becton–Dickinson).

For PRDX2-knockdown, ORL-48T cells were seeded into 24-well plates at 150,000 cells per well. Thereafter, cells were transfected using Lipofectamine 2000 with either 50 or 100 nM siRNA-Control or siRNA-PRDX2 according to manufacturer’s instructions. Sixteen hours after siRNA transfection, the medium was changed to DMEM/F12 containing 2.5% FBS and maintained for 72 hours as above.

### Cell migration in PRDX2-overexpressing cells and siRNA knockdown

PRDX2-overexpressing ORL-48T cells were seeded into 24-well tissue-culture plates at a density of 150,000 cells per well. After incubation for 24 hours, a linear gap was created by scratching the bottom of each well using a SPLScar Scratcher (SPL Life Science, Korea). The cells were then maintained in DMEM/F12 medium supplemented with 1% FBS and antibiotics for 24 and 48 hours. The extent of wound closure was determined using NIS-Elements Advanced Research Imaging Software version 3.0. This was converted into a percentage that was relative to the initial width of the wound. Three independent replicates of each experiment were performed.

For PRDX2-knockdown, ORL-48T cells were seeded into 24-well plates at 150,000 cells per well. Thereafter, cells were transfected using Lipofectamine 2000 with either 100 nM siRNA-Control or 100 nM siRNA-PRDX2 according to manufacturer’s instructions. After transfection, cells were incubated overnight, then cells were maintained in DMEM/F12 medium supplemented with 1% FBS and antibiotics and prepared as above.

### Statistical analysis

Data are expressed as mean ± SEM (standard error of the mean). The symbols *, ** and *** denote statistically significant differences as *P* < 0.05, 0.01 and 0.001, respectively. All statistical analysis was performed using Prism5 software (GraphPad, San Diego, CA, USA). Comparisons between groups were made by Student’s t-tests or One-way ANOVA analysis.

## Results

### Demographic data

Cancer-free control participants and OSCC patients had average ages of 60 and 61 years, respectively. There were more females than males in both groups. HPV prevalence was 10.6% (8/75) in cancer-free controls and 25.3% (19/75) in OSCC patients. A history of betel quid chewing was reported by 8.0% (6/75) and 44% (33/75) of cancer-free controls and OSCC patients, respectively (S2 Table).

### Expression levels of PRDX2 in oral cell samples

**Fig 1:**
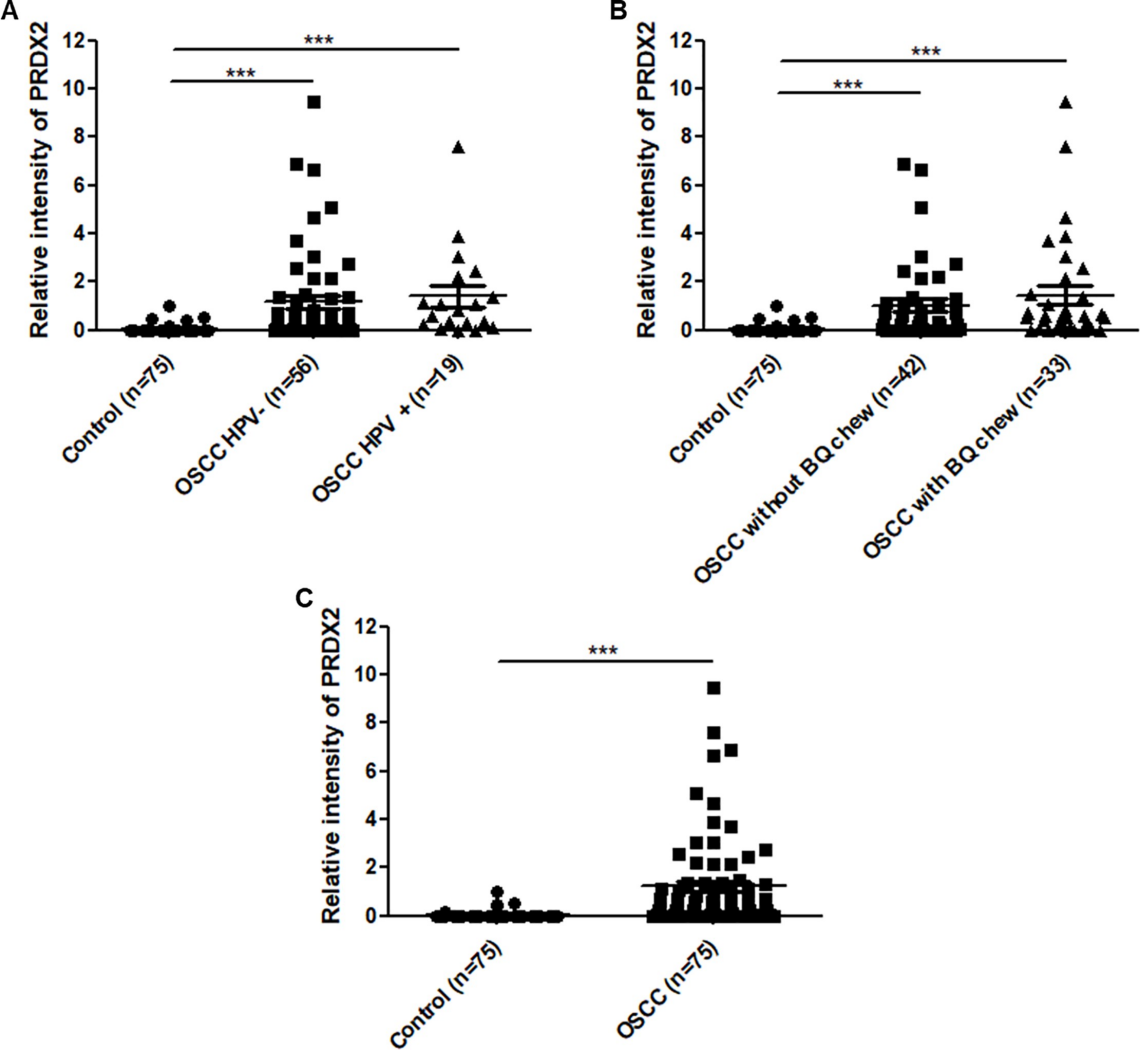
Relative intensity of PRDX2 in OSCC with/without HPV infection, or history of betel quid chewing. PRDX2 protein was detected by western blot in either exfoliated normal oral cells (cancer-free control; n = 75) or lesion cell samples with HPV-positive (n = 19) and negative (n = 56) OSCCs (A), and history of betel quid chewing (n = 33) and non-chewing (n = 42) (B). In addition, this protein was evaluated to compare between cancer-free control (n = 75) and OSCC (n = 75) groups (C). Beta-actin was used as an internal control. One dot in the scatter plot represents relative intensity of PRDX2 of a single sample. Error bars in the scatter plots represent the mean with standard error of the mean (SEM). Mann-Whitney test was used to compare expression levels. *** denotes statistically significant differences as *P* < 0.001.

To assess the correlation between HPV infection and PRDX2 protein expression in OSCC patients, relative intensity of PRDX2 protein was compared between HPV-negative and HPV-positive oral cell lesion samples. Relative intensity of PRDX2 in HPV-positive samples (1.39 ± 0.43) was slightly higher than in HPV-negative samples (1.16 ± 0.26), but the difference was not statistically significant (Fig 1A). Similarly, in OSCC samples from individuals with a history of betel quid chewing, the relative intensity of PRDX2 expression was higher (1.44 ± 0.38) than in samples from patients with no such history (1.02 ± 0.26) (Fig 1B), but without statistical significance. We note that the level of PRDX2 expression in these OSCC categories was significantly higher than among cancer-free controls (Fig 1C). This suggests that upregulation of PRDX2 is associated with OSCC and that HPV infection and betel quid chewing may partially affect its expression.

### Upregulation of PRDX2 in HPV16 E6/E7-expressing cells

**Fig 2:**
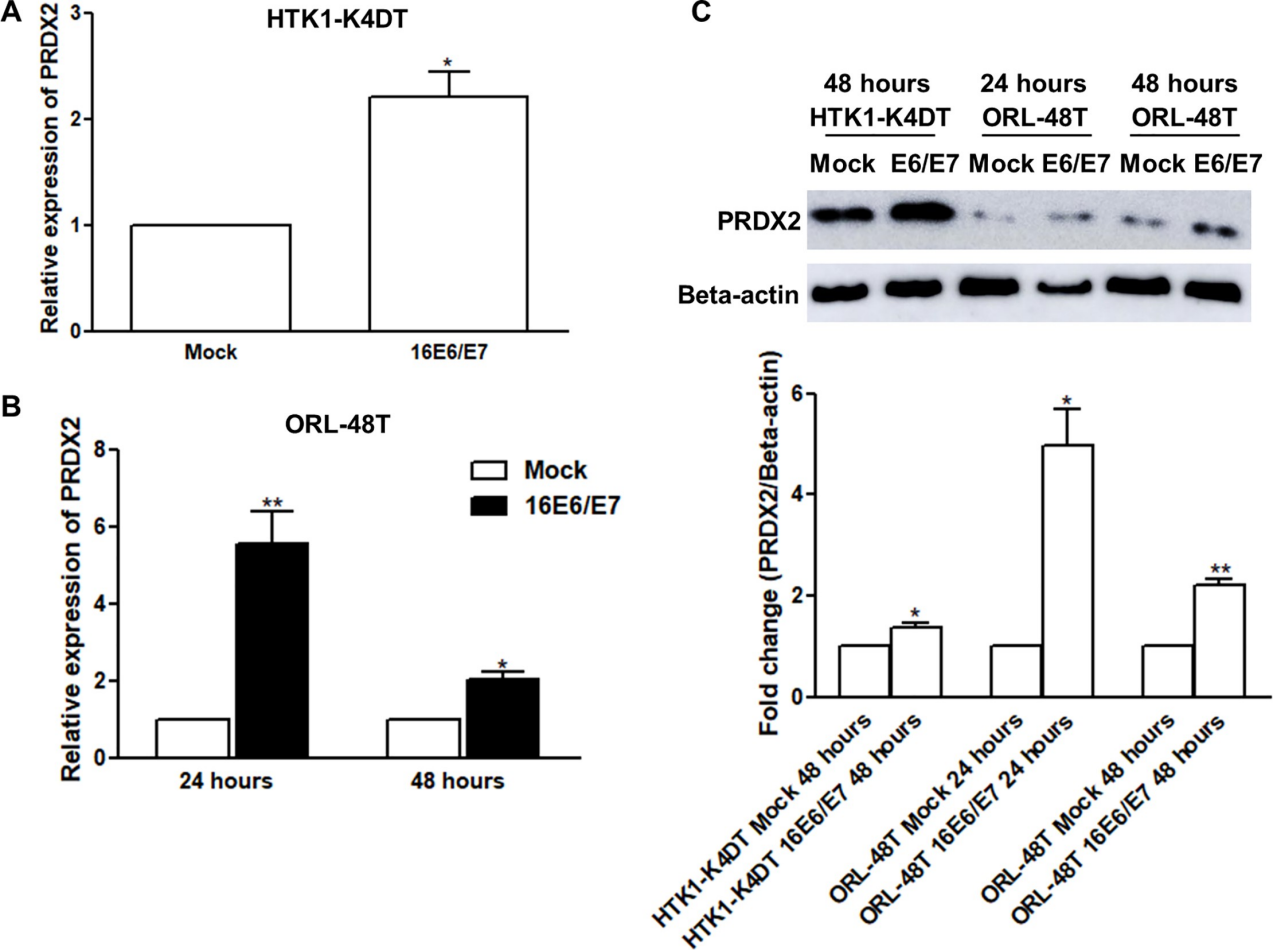
The level of PRDX2 mRNA and protein in HTK1-K4DT and ORL-48T cells transiently transfected with pIRES2-16E6/E7. Levels of PRDX2 mRNA and protein in mock (pIRES2)-transfected and HPV16 E6/E7 (pIRES2-16E6/E7)-transfected HTK1-K4DT (the immortalized human tongue keratinocyte) cells (A) and ORL-48T (well differentiated SCC that originated from mouth/gum) cells (B) was determined by Real-Time PCR and western blot. Each experiment was performed in triplicate. The protein intensity was measured using ImageJ 1.49v software. The protein intensity of PRDX2 was normalized with beta-actin to calculate the fold change (C). Differences in expression between mock and HPV16 E6/E7 treatments were statistically analyzed using paired t-tests. * and ** denote statistically significant differences as *P* < 0.05 and 0.01, respectively.

To examine PRDX2 expression *in vitro*, HPV16 E6/E7-transfected HTK1-K4DT and ORL-48T cells were investigated. We found that the overexpression of HPV16 E6/E7 led to upregulation of PRDX2 mRNA and protein compared with empty vector-transfected cells as shown in Fig 2. We also investigated the effect of HPV16 E6 on PRDX2 expression in HTK1-K4DT-16E6SD cells and found that PRDX2 mRNA and protein were upregulated in HTK1-K4DT-16E6SD relative to HTK1-K4DT (S1 Fig).

### Upregulation of PRDX2 in arecoline-treated oral cell lines

**Fig 3:**
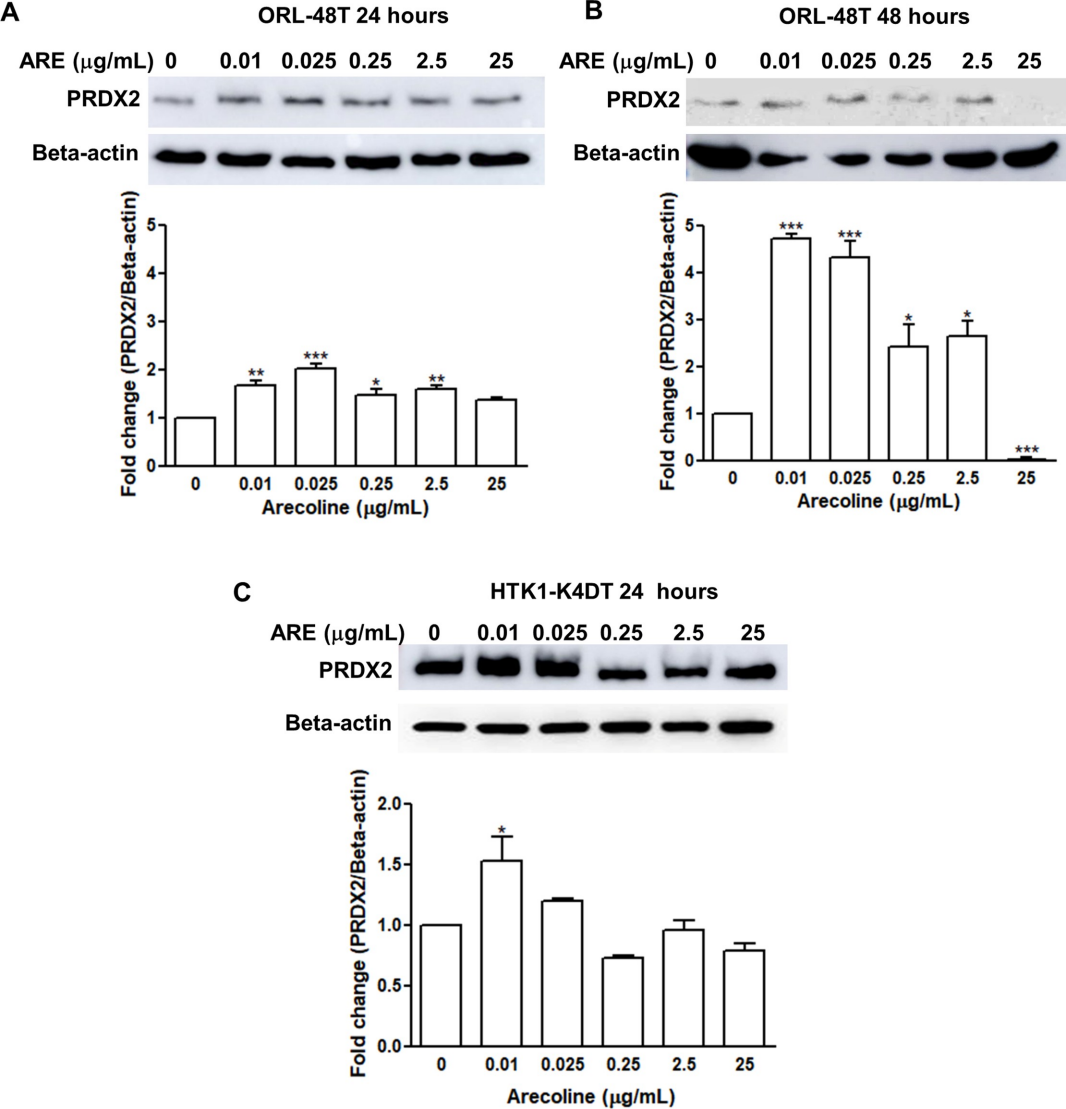
The level of PRDX2 protein expression in arecoline-treated oral cell lines. Oral cell lines, ORL-48T were treated with arecoline for 24 (A) and 48 (B) hours, and HTK1-K4DT for 24 (C) hours. Each experiment was performed in triplicate. The levels of PRDX2 protein was assessed by western blot. Beta-actin was used as an internal control. The protein intensity was measured using ImageJ 1.49v software. Fold change in protein intensity of PRDX2 was quantified. Differences in expression among 0–25 μg/ml of arecoline treatment were statistically analyzed using One-way ANOVA. * and ** denote statistically significant differences as *P* < 0.05 and 0.01, respectively.

To assess the effect of arecoline on PRDX2 expression, ORL-48T and HTK1-K4DT cells were treated with arecoline at various concentrations. The level of PRDX2 protein in arecoline-treated cells after 24 and 48 hours was higher than in untreated cells (Fig 3A–3C). The highest expression of PRDX2 in ORL-48T cells was found at concentrations of arecoline between 0.01 and 0.025 μg/mL. After 48 hours, the intensity of the PRDX2 band was higher than in untreated in ORL-48T cells at all concentrations of arecoline except 25 μg/mL (Fig 3B). In HTK1-K4DT cells, only arecoline concentrations of 0.01 and 0.025 μg/mL led to higher expression levels of PRDX2 than in untreated cells (Fig 3C). This indicates that lower concentrations of arecoline are more effective in upregulation of PRDX2 expression.

### PRDX2 promotes cell proliferation and cell-cycle progression

**Fig 4:**
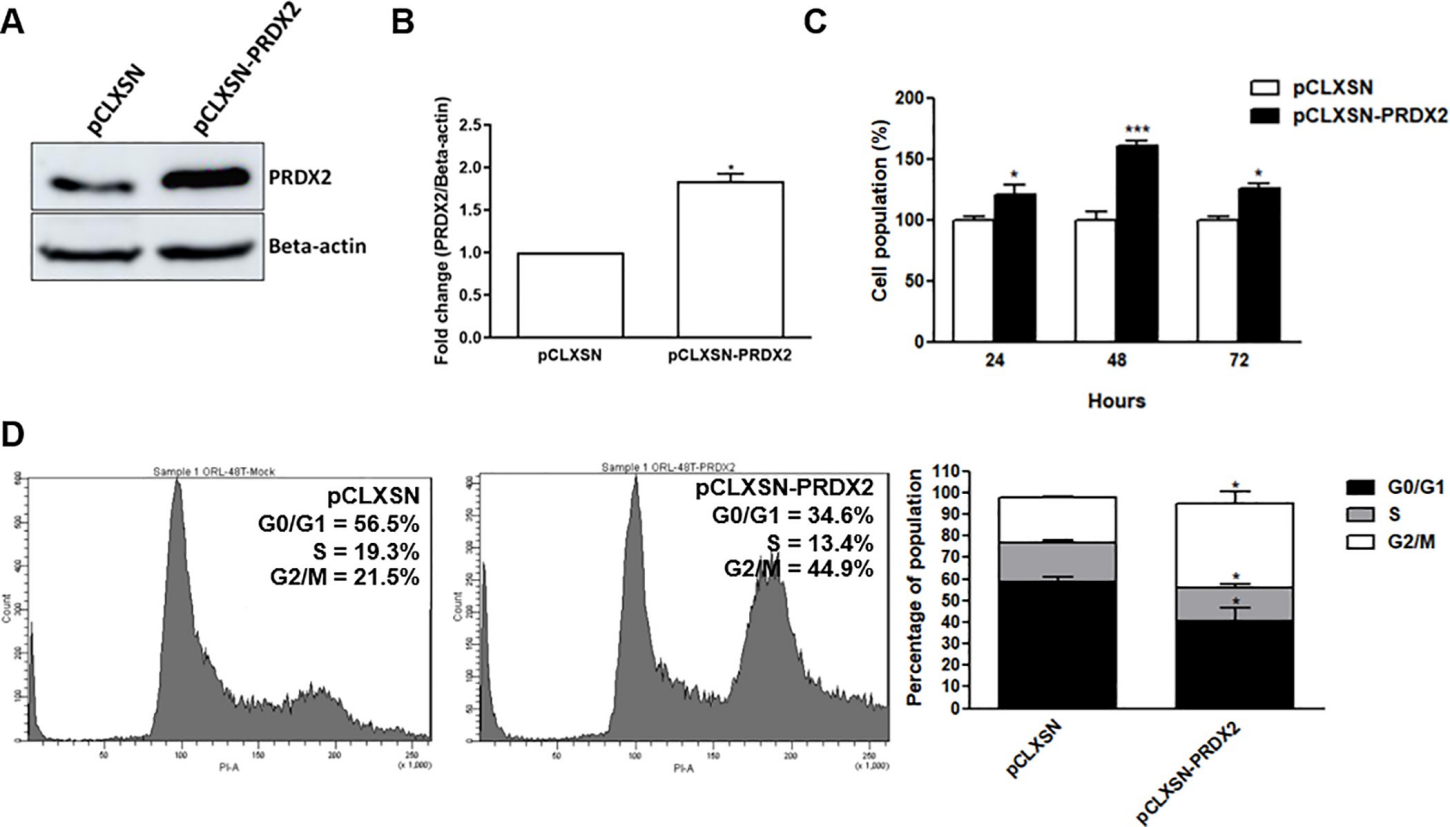
The effect of PRDX2 overexpression on cell proliferation and cell-cycle progression of ORL-48T cells. The level of PRDX2 protein in pCLXSN-PRDX2-transduced ORL-48T cells (A and B) was observed using western blot. Percentage of population of PRDX2-overexpressing ORL-48T cells relative to controls after incubation for 24, 48 and 72 hours was investigated by MTT assay (C). Differences in expression between pCLXSN and pCLXSN-PRDX2 were statistically analyzed using paired t-tests of triplicate experiments. Cell-cycle phase distribution in PRDX2-overexpressing ORL-48T cells incubated for 72 hours was determined by flow cytometry (D). Differences in expression and cell-cycle phase populations between pCLXSN and pCLXSN-PRDX2 were statistically analyzed using paired t-tests of triplicate experiments. * and *** denote statistically significant differences as *P* < 0.05 and 0.001, respectively.

To study the effect of PRDX2 on cell proliferation, percentage of cell population in PRDX2-overexpressing cells (pCLXSN-PRDX2) (Fig 4A and 4B) was assessed using the MTT assay. The proportion of these cells viable at 24, 48 and 72 hours (120.9% ± 18.8, 160.8% ± 5.8 and 125.3% ± 7.4, respectively) was significantly higher than control cells (pCLXSN-expressing cells) (Fig 4C). Concordantly, cell population (74.8% ± 6.5) of PRDX2-knockdown cells at 48 hours was significantly reduced when compared to siR-control-transfected cells (S2A–S2C Fig). A similar result was also found in PRDX2-knockdown HTK1-K4DT and ORL-136T cells (S2D and S2E Fig). This suggests that PRDX2 has a role in proliferation of OSCC cells.

The effect of PRDX2 on cell-cycle progression was investigated. The proportion of PRDX2-overexpressing cells in the G2/M phase was significantly higher (35.95% ± 12.6) and the proportion in the G0/G1 (40.6 ± 10.4) and S (15.2 ± 3.1) phases was lower than in control cells (18.7% ± 4.0, 58.6 ± 3.6, and 18.3 ± 2.0, respectively) (Fig 4D). In contrast, in PRDX2 knockdown ORL-48T cells, the G2/M proportion (22.2% ± 1.3) was lower than controls (S2F Fig). This result might indicate that PRDX2 promotes cell-cycle progression at the G2/M population.

### PRDX2 promotes cell migration in ORL-48T cell lines

**Fig 5:**
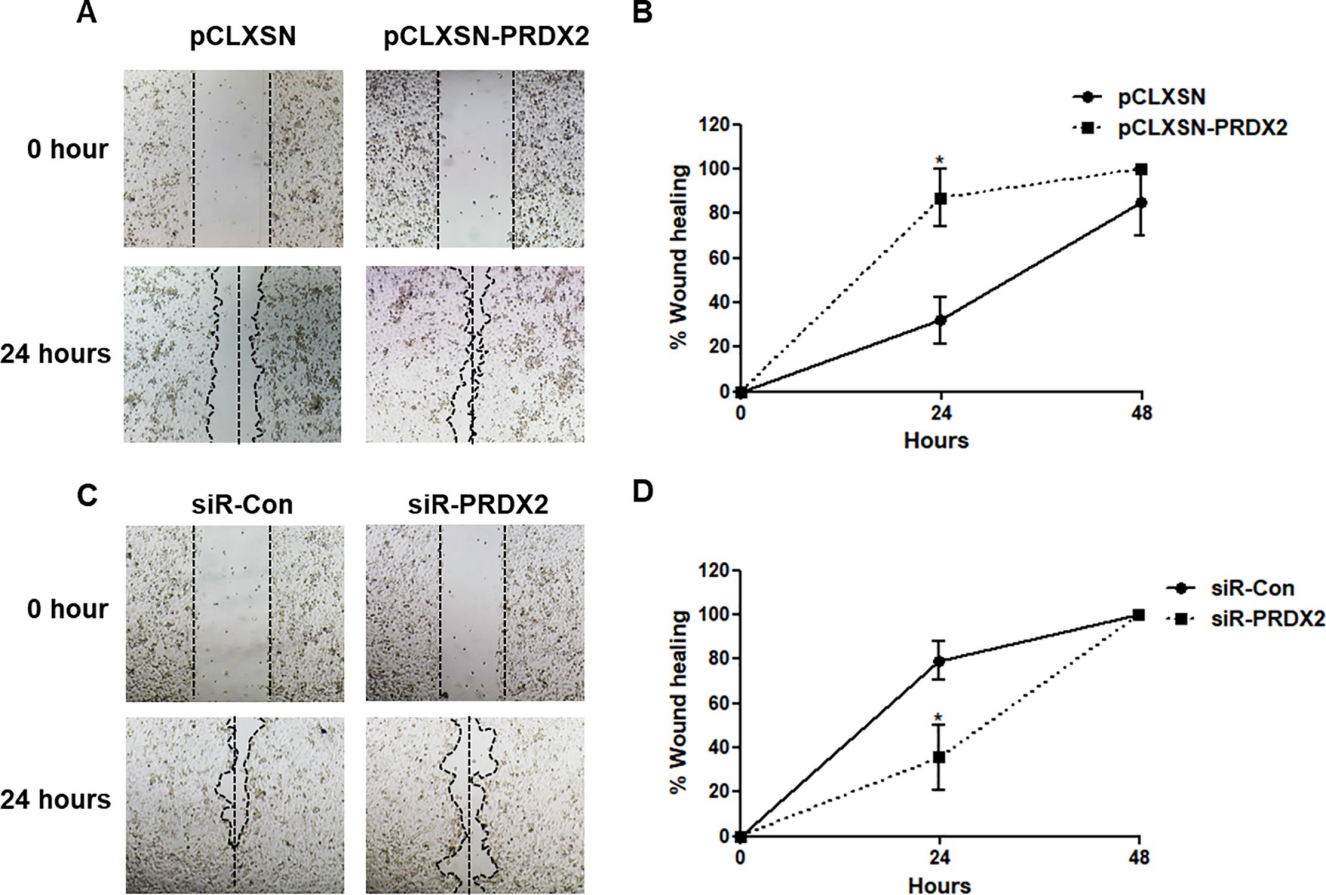
Cell migration in PRDX2-overexpressing cells and knockdown ORL-48T cells using a wound-healing assay. ORL-48T transduced with retrovirus-carrying pCLXSN or pCLXSN-PRDX2 (A and B) and transfected with siRNA-Control (siR-Con) and siR-PRDX2 (C and D) were maintained in DMEM/F12 medium with 1% FBS. After incubation for 24 hours, a wound was created and measured at 0, 24 and 48 hours using NIS-Elements Advanced Research Imaging Software version 3.0 and the 4x objective lens of an inverted microscope. Statistical comparisons were done using paired t-tests of triplicate experiments. * denotes statistically significant differences as *P* < 0.05.

To determine the effect of PRDX2 on cell migration, ORL-48T cells transduced with pCLXSN or with pCLXSN-PRDX2 were cultured in DMEM/F12 medium. A linear gap was created by scratching the cell layer on the bottom of the dish. The cells were then maintained in 1% FBS medium for 24 and 48 hours. The extent of wound closure was determined. After 24 hours, wound closure by PRDX2-overexpressing ORL-48T cells was significantly greater (87.0% ± 22.4) than by control cells (32.0% ± 18.4) (Fig 5A and 5B). In knockdown cells with siRNA-control and siRNA-PRDX2 cells, wound healing rates were 79.1% ± 17.3 and 35.5% ± 29.4, respectively (Fig 5C and 5D). The result showed that PRDX2 can promote cell migration in ORL-48T cells.

### PRDX2 inhibits apoptosis

**Fig 6:**
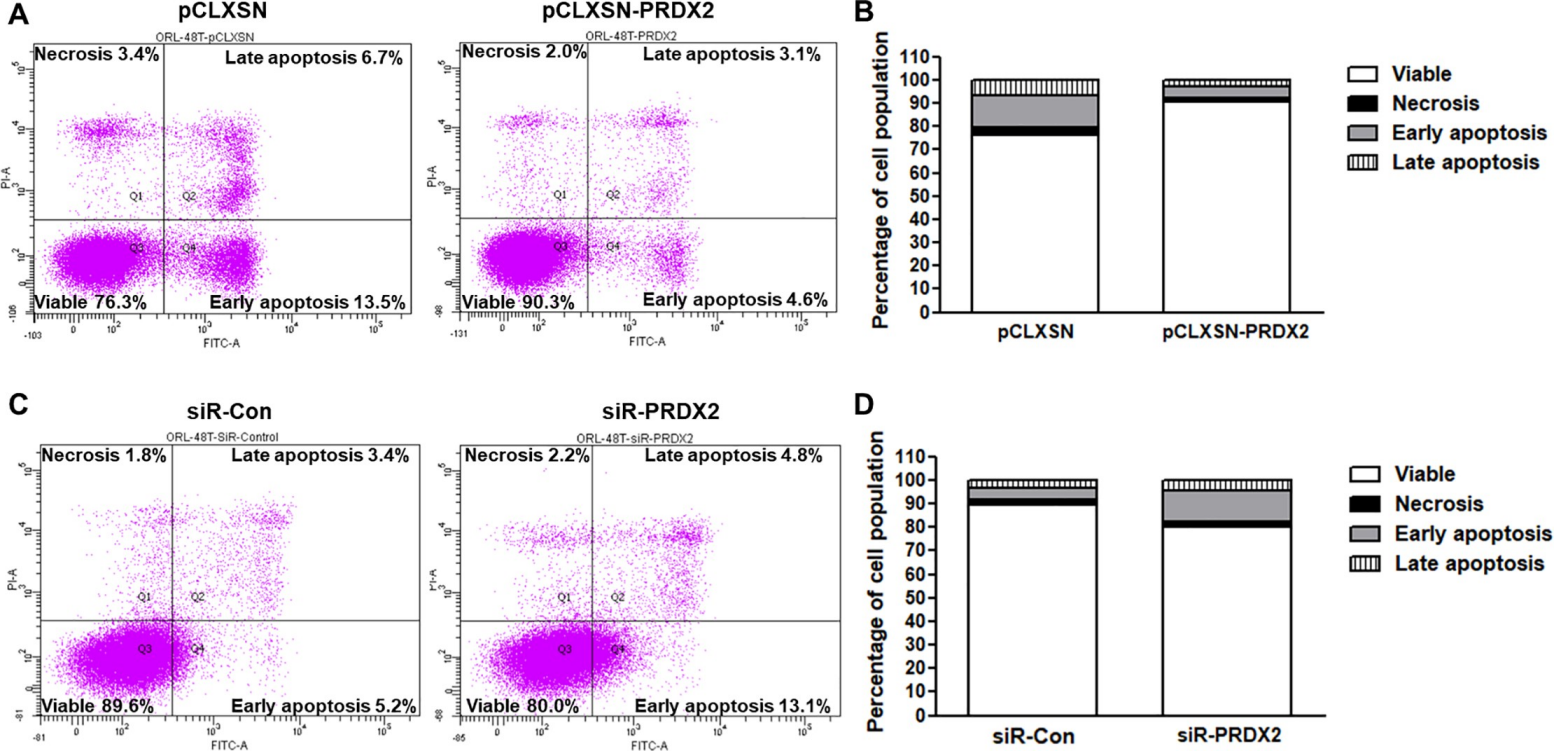
PRDX2-overexpressing and siR-PRDX2 knockdown ORL-48T cells in apoptosis analysis. ORL-48T transduced with retrovirus-carrying pCLXSN or pCLXSN-PRDX2 (A and B) and transfected with siRNA-Control (siR-Con) and siR-PRDX2 (C and D) were maintained in DMEM/F12 medium supplemented with 2.5% FBS for 72 hours. The analysis of apoptosis in pCLXSN-PRDX2-transduced (A and B) and siR-PRDX2-transfected (C and D) ORL-48T cells was performed using flow cytometry with an apoptosis kit.

The effect of PRDX2 on apoptosis was examined using flow cytometry with an apoptosis kit. A smaller proportion of PRDX2-overexpressing ORL-48T cells were apoptotic (4.6%), particularly in early apoptosis, relative to controls (13.5%) (Fig 6A and 6B). Conversely, PRDX2-knockdown ORL-48T cells had a greater proportion of early apoptotic cells (Fig 6C and 6D). This result demonstrates that PRDX2 functions as an inhibitor of apoptosis in OSCC.

## Discussion

We investigated the role of HPV oncoprotein and arecoline on PRDX2 expression in OSCC cells. Additionally, the function of PRDX2 in cell proliferation, cell-cycle progression, cell migration and apoptosis in oral cell lines was examined.

Overexpression of PRDX2 is a potential biomarker for colorectal cancer [27], OSCC [28], osteosarcoma [29] and ovarian cancer [30]. However, the induction and role of PRDX2 expression in OSCC remain unclear. Unsurprisingly, we found that PRDX2 acts as a tumor promoter involved in the induction of cell proliferation, cell-cycle progression and inhibition of apoptosis in oral cell lines. Consistently, PRDX2 functions as a tumor promoter in colorectal cancer [5]. Importantly, PRDX2 has a direct role in lung metastasis, and high expression of PRDX2 in breast cancer is associated with progression to lung metastasis [7]. High expression of PRDX2 is significantly associated with ovarian cancer progression including pathological grades II and III, and clinical stages III and IV, as well as with chemotherapeutic drug resistance [30]. These studies demonstrated the roles of PRDX2 in advanced stages of various malignancies. Interestingly, we found that PRDX2 promoted cell migration in ORL-48T (derived from an advanced stage of OSCC) but not in ORL-136T cells (derived from early-stage OSCC tissue) or in HTK1-K4DT cells (an immortalized tongue keratinocyte cell line derived from normal keratinocytes) (S3 Fig) [31]. Additionally, the population of G2/M was significantly higher in PRDX2-overexpressing cells than in control cells that interpreting as cell-cycle progression more than cell-cycle arrest because we found the upregulation of c-Myc and downregulation of p16^INK4A^ which functioned as an induction of cell-cycle progression and of arrest respectively in compared with control cells (S4 Fig) [32–35]. However, cell-cycle arrest cannot be ruled out in PRDX2-overexpressing cells [36]. These observations suggest that PRDX2 may play a role in promotion of OSCC progression, particularly in advanced stages of disease. The mechanism of PRDX2 exerting its oncogenic action in various malignancies is still poorly understood; therefore, additional studies are needed to further investigate this mechanism. We hypothesized that PRDX2 act as a tumor promoter via neutralizing hydrogen peroxide contributing to the protection of cells from oxidative damage and the regulation of peroxide-mediated signal transduction in oral cell lines. These molecular mechanisms influence other signaling cascades including NF-κB, c-Myc and cytochrome release that may promote tumorigenesis [7]. Wu et al. demonstrated that decreased PRDX2 exerted cell-cycle arrest and apoptosis and inhibited proliferation in trophoblast via ROS-related p-p53 and p38-MAPK/p21 pathway [37]. Feng et al. indicated the oncogenic role of PRDX2 in esophageal squamous cell carcinoma via Wnt/β-catenin and AKT pathways [38].

Even though PRDX2 upregulation can be a potential biomarker in various cancers as mentioned above, how it is upregulated and its role in OSCC development remains obscure. This study investigated the effect of arecoline and HPV oncogenes on PRDX2 in both mRNA and protein expression level. As expected, both arecoline treatment (low concentration) and HPV16 E6/E7 could induce PRDX2 overexpression in oral cell lines including oral cancer (ORL-48T and ORL-136T) and immortalized tongue keratinocyte (HTK1T-KTD4) cell lines. Conversely, high concentration of arecoline treatment reduced PRDX2 expression (Fig 3B and 3C) and induced cell death (S5 Fig) at 48 hours.

The expression of PRDX2 is mainly induced by oxidative stress [39]. Oxidative stress induces PRDX2 expression via the CD36 signaling cascade, leading to activation of transcription factor Nrf2 to bind specific ARE elements; subsequently key antioxidant genes are transcribed [12]. In another way, epigenetics also regulates PRDX2 expression [10, 11]. Unsurprisingly, arecoline promotes oxidative stress [40] and modulates epigenetic alteration [41]. Supporting this study, arecoline induces PRDX2 expression in oral cell lines. In addition, Lin et al. suggested that arecoline contributes changes in the expression of several genes catalyzing histone methylation, acetylation, and demethylation [41]. In addition, c-Myc induced in oral cell lines underlying arecoline treatment could bind to PRDX2 promoter and regulate PRDX2 expression by various pathways [10, 25, 37, 40]. Our previous study also found a role of this compound in downregulating miRNA in oral cancer [25]. In the other hand, there are reports of oxidative stress being induced by HPV16 E6, particularly the truncated splice variant (E6*) [42]. HPV E6 and E7 also induced HIF-1α and Nrf2 [21, 43]. In addition, HPV E6/E7/Notch could upregulate c-Myc [44]. These mechanisms by which HPV oncogenes may link to PRDX2 regulation. Infection with this virus also regulates epigenetics [45]. Moreover, we also predicted transcription factor binding sites upstream promoter region of *PRDX2* gene using GeneCards® (https://www.genecards.org/) and subsequently found that the promoter contained c-Fos, JunD, JunB, c-Myc, E2F, STAT3, DNMT3b, SP1 and YY1 binding sites (S6 Fig). These transcription factors are involved in the regulatory network of either arecoline or HPV oncoprotein. It is possible that arecoline and HPV may regulate PRDX2 through those mechanisms.

In summary, PRDX2 acts as a tumor promoter in oral cell lines and its dysregulation could be induced by arecoline and HPV16 E6/E7.

## Supporting information

S1 Fig The expression levels of PRDX2 protein and mRNA in HTK1-K4DT-16E6SD cells and in HTK1-K4DT cells. Expression of HPV16 E6 (A) and PRDX2 (B) mRNA in HTK1-K4DT and HTK1-K4DT-16E6SD cells incubated for 24 and 48 hours was examined by Real-Time PCR. PRDX2 protein (C) in HTK1-K4DT and HTK1-K4DT-16E6SD cells incubated for 24 and 48 hours was detected by western blot. The experiments were performed in triplicate.
(TIF)

S2 Fig The effect of PRDX2-knockdown on cell proliferation and cell-cycle progression of OSCC cells. PRDX2 protein levels following PRDX2 knockdown in siR-PRDX2-transfected ORL-48T cells (siR-PRDX2) was compared with controls (siR-Con) by western blot (A and B). Percentage of cell population in PRDX2-knockdown ORL-48T (C), HTK1-K4DT (D), and ORL-136 (E) cells at 48 hours post-transfection was determined using the MTT assay. Cell-cycle phase distribution in PRDX2-knockdown ORL-48T cells incubated for 72 hours was determined by flow cytometry (F). Each experiment was performed in triplicate.
(TIF)

S3 Fig Cell migration in PRDX2-knockdown ORL-136T and HTK1-K4DT cells using a wound-healing assay. ORL-136T (A and B) and HTK1-K4DT (C and D) transfected with siRNA-Control (siR-Con) and siR-PRDX2 (C and D) were maintained in DMEM/F12 with 1% FBS and F medium with 0.5%, respectively. After incubation for 24 hours, a wound was created and measured at 0, 24 and 48 hours using NIS-Elements Advanced Research Imaging Software version 3.0 and the 4x objective lens of an inverted microscope. Experiments were performed in triplicate.
(TIF)

S4 Fig The level of p16^INK4A^ and c-Myc mRNAs in PRDX2-overexpressing ORL-48T cells. PRDX2-overexpressing ORL-48T cells were seeded into 24-well plates at a density of 150,000 cell per well. After incubation for 24 hours, the medium was changed to DMEM/F12 containing 2.5% FBS and antibiotics and maintained for 72 hours. Total RNA was extracted and used to cDNA synthesis. The level of p16^INK4A^ (A) and c-Myc (B) mRNAs was analyzed by Real-Time PCR. GAPDH was used as an internal control. Experiments were performed in triplicate.
(TIF)

S5 Fig MTT assay for determination of cell cytotoxicity in arecoline-treated ORL-48T and HTK1-K4DT cells. Either ORL-48T (A) or HTK1-K4DT (B) cell line was seeded into each well of 96-well plate at a density of 2 x 10^4^ cells per well. After 24 hours of time incubation, the cells were treated with various concentrations of arecoline and incubated for 48 hours. Cell viability was analyzed by MTT reagent. Experiments were performed in triplicate and repeated three times.
(TIF)

S6 Fig Promoter and enhancer for *PRDX2* gene in GeneCards. A list of transcription factors having transcription factor binding sites within the PRDX2 promoter and enhancer are presented in blue letter. Red box represents transcription factors regulated by arecoline and/or HPV. The table describes a candidate promoter and enhancer associated with the *PRDX2* gene. An asterisk represents confidence score that has elite promoters and enhancers.
(TIF)

S1 Table Primer sequences. (DOCX)

S2 Table Demographic data of cancer-free controls and OSCC patients. (DOCX)

S1 Raw images (PDF)
