# Supplementary figures and images for: Peroxiredoxin 2 is highly expressed in human oral squamous cell carcinoma cells and is upregulated by human papillomavirus oncoproteins and arecoline, promoting proliferation
Source: PLoS One. 2020 Dec 17;15(12):e0242465. doi: 10.1371/journal.pone.0242465 (PMC7746188; doi:10.1371/journal.pone.0242465)

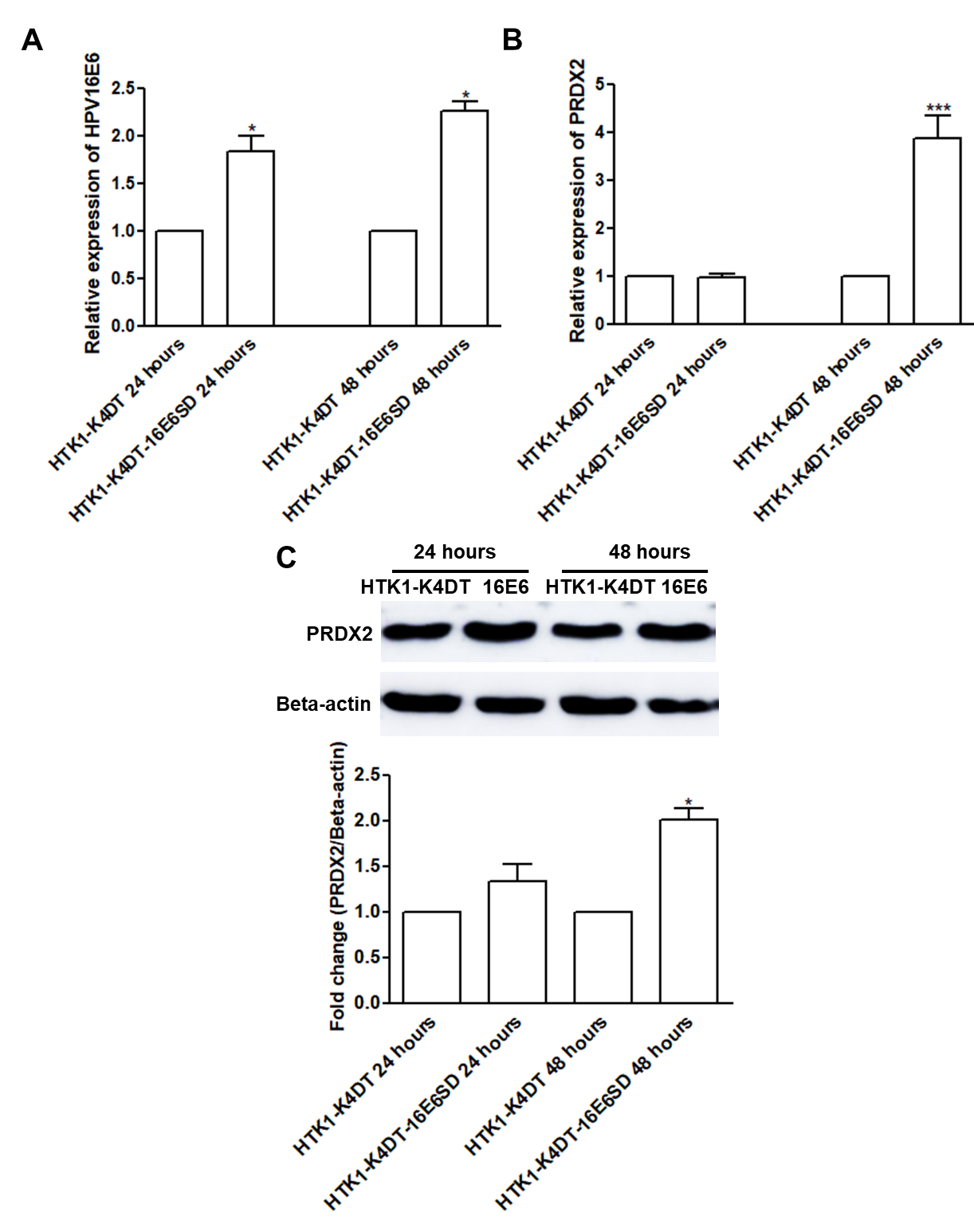

Supplement: S1 Fig — Expression of HPV16 E6 (A) and PRDX2 (B) mRNA in HTK1-K4DT and HTK1-K4DT-16E6SD cells incubated for 24 and 48 hours was examined by Real-Time PCR. PRDX2 protein (C) in HTK1-K4DT and HTK1-K4DT-16E6SD cells incubated for 24 and 48 hours was detected by western blot. The experiments were performed in triplicate. (TIF) [file pone.0242465.s001.tif]

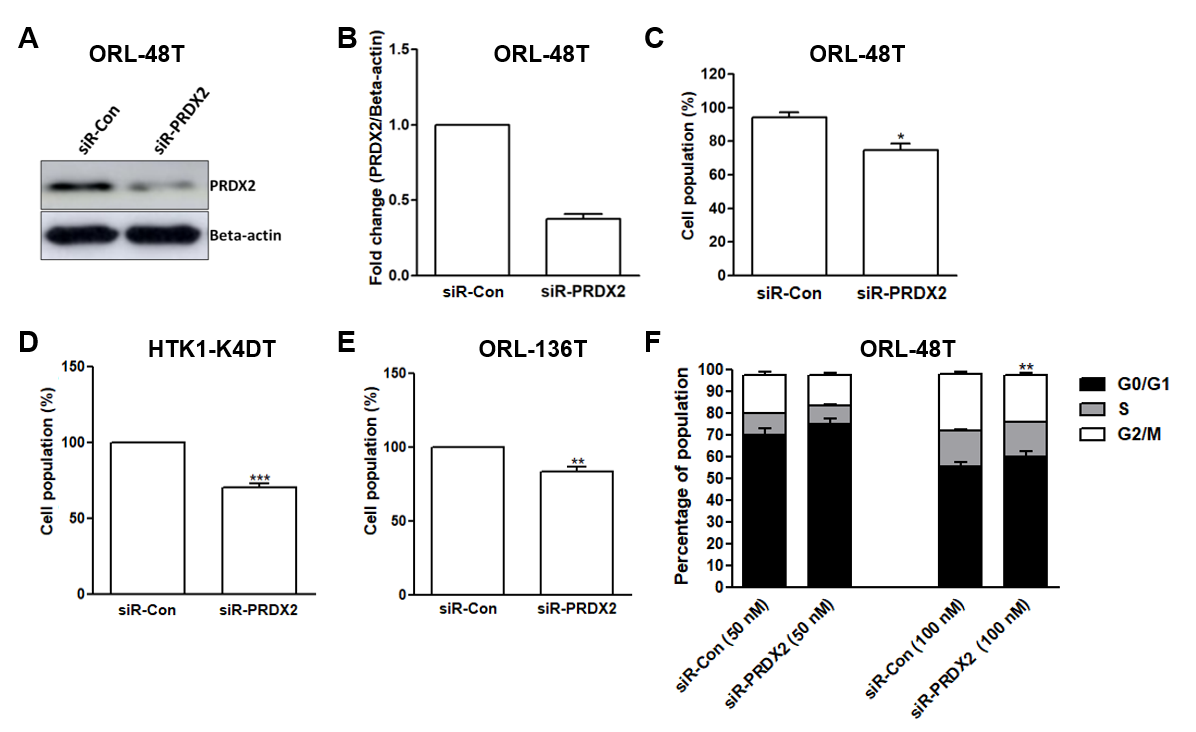

Supplement: S2 Fig — PRDX2 protein levels following PRDX2 knockdown in siR-PRDX2-transfected ORL-48T cells (siR-PRDX2) was compared with controls (siR-Con) by western blot (A and B). Percentage of cell population in PRDX2-knockdown ORL-48T (C), HTK1-K4DT (D), and ORL-136 (E) cells at 48 hours post-transfection was determined using the MTT assay. Cell-cycle phase distribution in PRDX2-knockdown ORL-48T cells incubated for 72 hours was determined by flow cytometry (F). Each experiment was performed in triplicate. (TIF) [file pone.0242465.s002.tif]

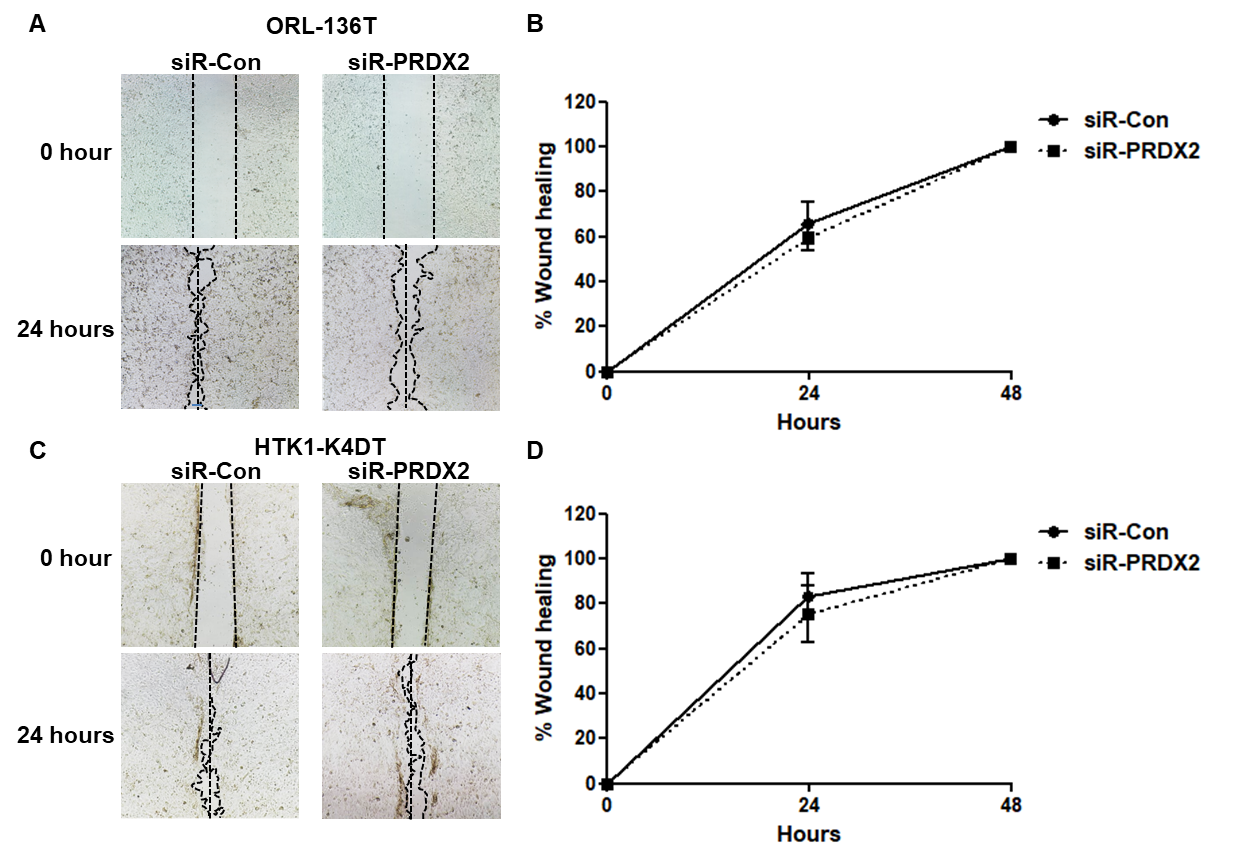

Supplement: S3 Fig — ORL-136T (A and B) and HTK1-K4DT (C and D) transfected with siRNA-Control (siR-Con) and siR-PRDX2 (C and D) were maintained in DMEM/F12 with 1% FBS and F medium with 0.5%, respectively. After incubation for 24 hours, a wound was created and measured at 0, 24 and 48 hours using NIS-Elements Advanced Research Imaging Software version 3.0 and the 4x objective lens of an inverted microscope. Experiments were performed in triplicate. (TIF) [file pone.0242465.s003.tif]

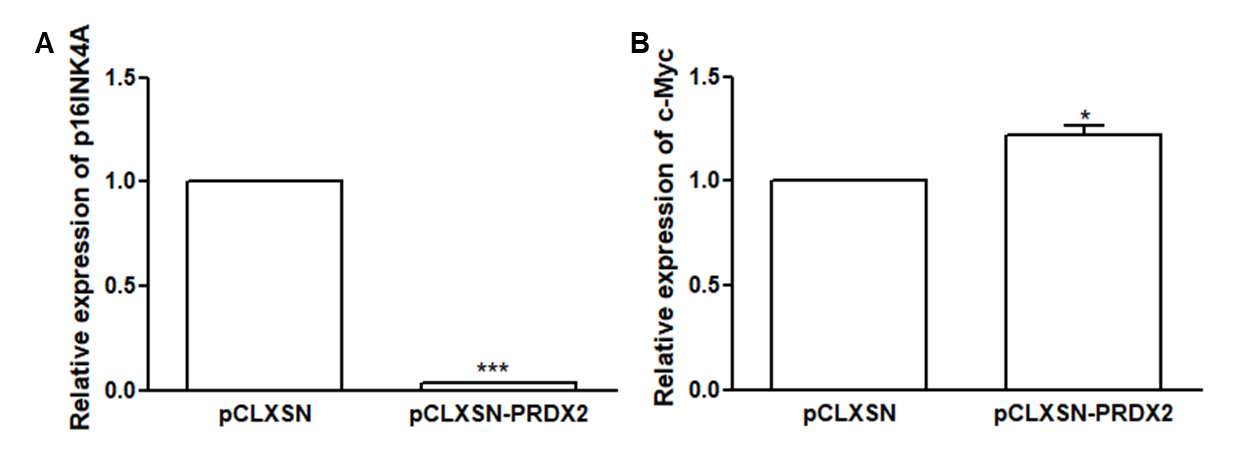

Supplement: S4 Fig — PRDX2-overexpressing ORL-48T cells were seeded into 24-well plates at a density of 150,000 cell per well. After incubation for 24 hours, the medium was changed to DMEM/F12 containing 2.5% FBS and antibiotics and maintained for 72 hours. Total RNA was extracted and used to cDNA synthesis. The level of p16INK4A (A) and c-Myc (B) mRNAs was analyzed by Real-Time PCR. GAPDH was used as an internal control. Experiments were performed in triplicate. (TIF) [file pone.0242465.s004.tif]

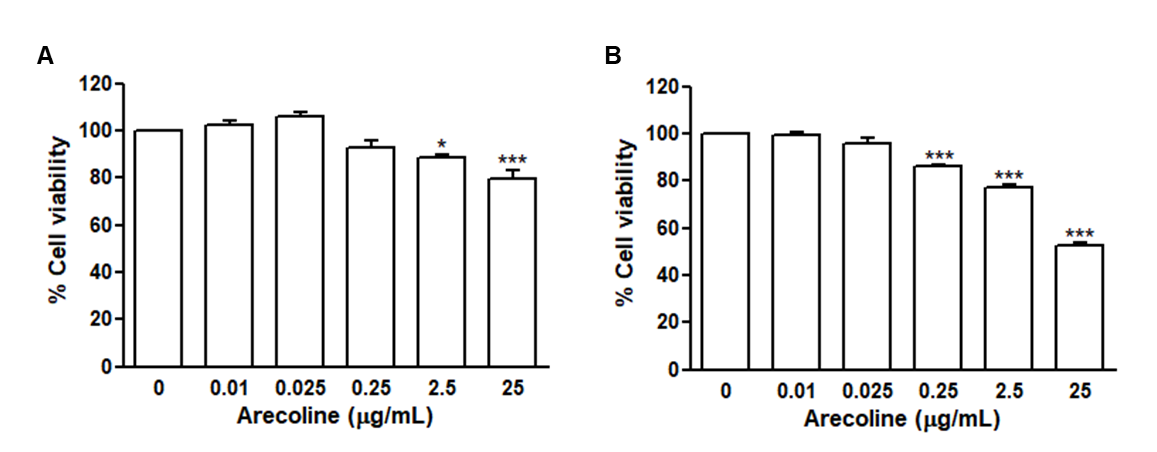

Supplement: S5 Fig — Either ORL-48T (A) or HTK1-K4DT (B) cell line was seeded into each well of 96-well plate at a density of 2 x 10^4^ cells per well. After 24 hours of time incubation, the cells were treated with various concentrations of arecoline and incubated for 48 hours. Cell viability was analyzed by MTT reagent. Experiments were performed in triplicate and repeated three times. (TIF) [file pone.0242465.s005.tif]

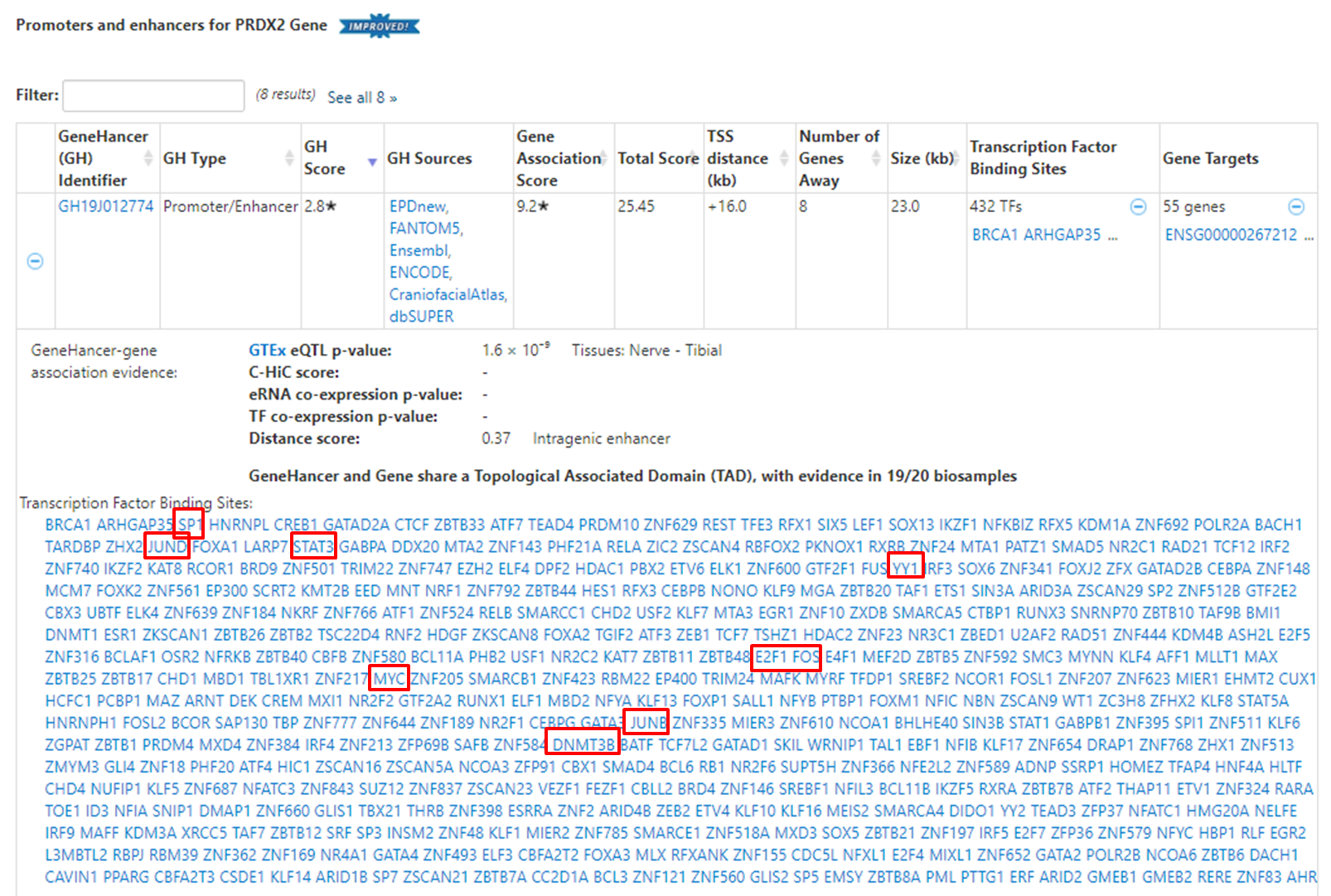

Supplement: S6 Fig — A list of transcription factors having transcription factor binding sites within the PRDX2 promoter and enhancer are presented in blue letter. Red box represents transcription factors regulated by arecoline and/or HPV. The table describes a candidate promoter and enhancer associated with the PRDX2 gene. An asterisk represents confidence score that has elite promoters and enhancers. (TIF) [file pone.0242465.s006.tif]
