# Supplementary material for: Peroxiredoxin 2 is highly expressed in human oral squamous cell carcinoma cells and is upregulated by human papillomavirus oncoproteins and arecoline, promoting proliferation
Source: PLoS One. 2020 Dec 17;15(12):e0242465. doi: 10.1371/journal.pone.0242465 (PMC7746188; doi:10.1371/journal.pone.0242465)
Supplement: S1 Table — (DOCX) [file pone.0242465.s007.docx]

| Primer | Oligonucleotide sequence | Size |
| --- | --- | --- |
| PRDX2-F attB1′  PRDX2-R attB2′ | 5′-AAAAAGCAGGCTATATGGCCTCCGGTAACGCGCGCAT-3′  5′-AGAAAGCTGGGTCGGTTAATTGTGTTTGGAGAAATA-3′ | 680 bp |
| attB1AP′  attB2AP′ | 5′-GGGGACAAGTTTGTACAAAAAAGCAGGCT-3′  5′-GGGGACCACTTTGTACAAGAAAGCTGGGT-3′ | - |
| PRDX2-F′  PRDX2-R′ | 5′-CACCTGGCTTGGATCAACACC-3′  5′-CAGCACGCCGTAATCCTCAG-3′ | 108 bp |
| GAPDH-F  GAPDH-R | 5′-TCATCAGCAATGCCTCCTGCA-3′  5′-TGGGTAGCAGTGATGGCA-3′ | 117 bp |
