# Supplementary material for: Peroxiredoxin 2 is highly expressed in human oral squamous cell carcinoma cells and is upregulated by human papillomavirus oncoproteins and arecoline, promoting proliferation
Source: PLoS One. 2020 Dec 17;15(12):e0242465. doi: 10.1371/journal.pone.0242465 (PMC7746188; doi:10.1371/journal.pone.0242465)
Supplement: S2 Table — (DOCX) [file pone.0242465.s008.docx]

| Parameters | Number of samples (n) | |
| --- | --- | --- |
|  | Cancer-free controls, n = 75 | Cancer, n = 75 |
| Age |  |  |
| Average  Range | 60 ± 16.1  23-84 | 61 ± 14.8  20-82 |
| Gender |  |  |
| Male  Female | 35  40 | 30  45 |
| History of betel quid chewing |  |  |
| No | 69 | 42 |
| Yes | 6 | 33 |
| HPV infection |  |  |
| Negative | 67 | 56 |
| Positive | 8 | 19 |
