# Supplementary material for: Peroxiredoxin 2 is highly expressed in human oral squamous cell carcinoma cells and is upregulated by human papillomavirus oncoproteins and arecoline, promoting proliferation
Source: PLoS One. 2020 Dec 17;15(12):e0242465. doi: 10.1371/journal.pone.0242465 (PMC7746188; doi:10.1371/journal.pone.0242465)
Supplement: S1 Raw images — (PDF) [file pone.0242465.s009.pdf]

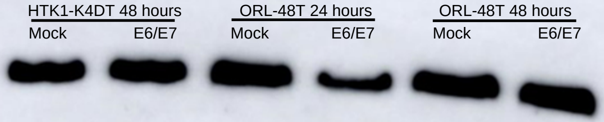

Fig 2 Beta-actin

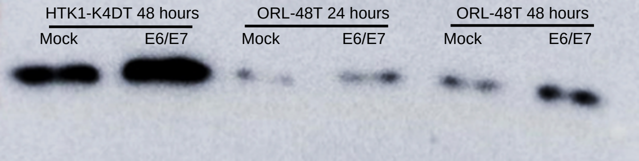
HTK1-K4DT 48 hours
ORL-48T 24 hours
ORL-48T 48 hours
Mock
E6/E7
Mock
E6/E7
Mock
E6/E7

Fig 2 PRDX2

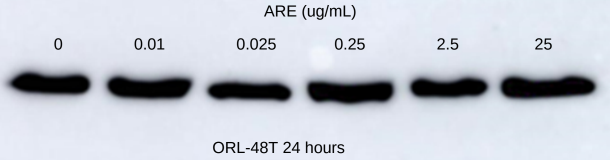

Fig 3 Beta-actin

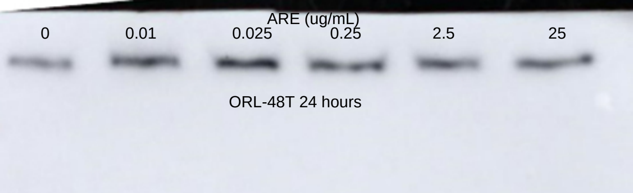

Fig 3 PRDX2

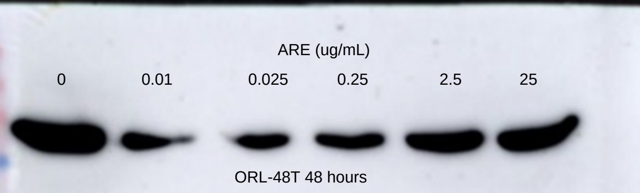

Fig 3 Beta-actin

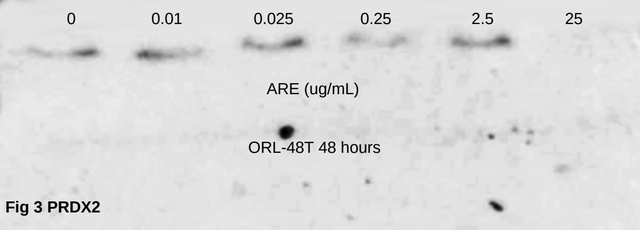

Fig 3 PRDX2

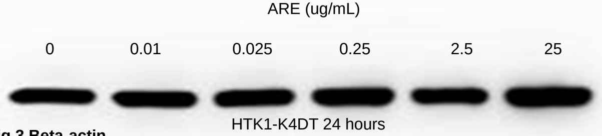

Fig 3 Beta-actin

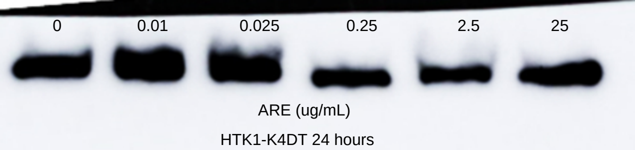

Fig 3 PRDX2

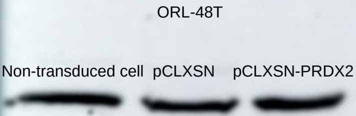

Fig 4 Beta-actin

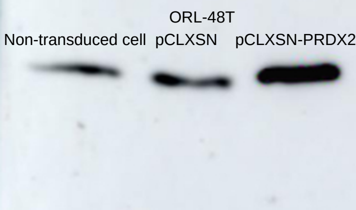

Fig 4 PRDX2

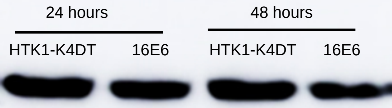

S1 Fig Beta-actin

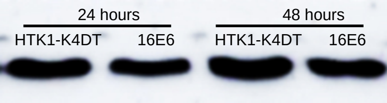

S1 Fig PRDX2

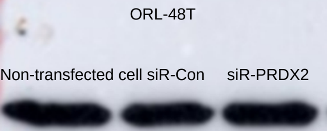

S2 Fig Beta-actin

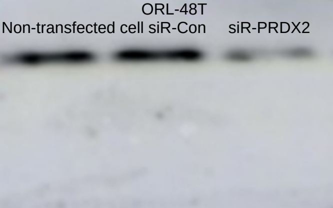

S2 Fig PRDX2
